# Differential Effects of High Sugar, High Lard or a Combination of Both on Nutritional, Hormonal and Cardiovascular Metabolic Profiles of Rodents

**DOI:** 10.3390/nu10081071

**Published:** 2018-08-11

**Authors:** Amanda Martins Matias, Wagner Müller Estevam, Priscila Murucci Coelho, Douglas Haese, Jéssika Butcovsky Botto Sarter Kobi, Ana Paula Lima-Leopoldo, André Soares Leopoldo

**Affiliations:** 1 Postgraduate Program in Nutrition and Health, Center of Health Sciences, Federal University of Espírito Santo, Vitória 29075-910, Espírito Santo, Brazil; matias.amandamartins@gmail.com (A.M.M.); priscilamurucci@hotmail.com (P.M.C.); j.butcovsky@gmail.com (J.B.B.S.K.); anapaulalimaleopoldo@gmail.com (A.P.L.-L.); 2 Postgraduate Program in Physical Education, Center of Physical Education and Sports, Federal University of Espírito Santo (UFES), Vitória 29075-910, Espírito Santo, Brazil; wagnerdvolei@hotmail.com; 3 University of Vila Velha, Vila Velha 29102-920, Espírito Santo, Brazil; douglas@ctapesquisas.com.br; 4 Department of Sports, Center of Physical Education and Sports, Federal University of Espírito Santo, Vitória 29075-910, Espírito Santo, Brazil

**Keywords:** obesity, comorbidities, hypercaloric diets, experimental model, rodents

## Abstract

Background: Dietary interventions in rodents can induce an excess of adipose tissue and metabolic disorders that resemble human obesity. Nevertheless, these approaches are not standardized, and the phenotypes may vary distinctly among studies. The aim of this study was to investigate the effects of different dietary interventions on nutritional, metabolic, biochemical, hormonal, and cardiovascular profiles, as well as to add to development and characterization of an experimental model of obesity. Methods: Male Wistar rats were randomized into four groups: control diet (C), high-sugar (HS), high-fat (HF), or high-sugar and high-fat (HFHS). Weekly measurements of body weight, adiposity, area under the curve (AUC) for glucose, blood pressure (BP) and serum triglycerides, total cholesterol level, and leptin were performed. Results: HF and HFHS models were led to obesity by increases in adipose tissue deposition and the adiposity index. All hypercaloric diets presented systolic BP increases. In addition, the AUC for glucose was greater in HF and HFHS than in C, and only the HF group presented hyperleptinemia. Conclusions: HF and HFHS diet approaches promote obesity and comorbidities, and thus represent a useful tool for studying human obesity-related disorders. By contrast, the HS model did not prove to be a good model of obesity.

## 1. Introduction

Obesity is a disease with multiple causes and one which is characterized by increased adipose tissue; it is considered a worldwide epidemic and a serious public health problem [1,2]. This complex disease is related to some disorders, such as dyslipidemias, insulin resistance, type 2 diabetes mellitus, certain cancers, and cardiovascular diseases [3]. Although it is clear that both genetic and environmental factors contribute to the propensity of an individual to be obese, authors emphasize that diet represents an important environmental factor that can influence obesity [4,5,6].

Animal models are commonly used to mimic human diseases and to improve understanding of the pathophysiology of obesity-related disorders [7]. Rodents which have the ability to develop obesity through genetic changes, such as leptin signaling defects *(ob*/*ob* mouse) or an autosomal recessive mutation in the fatty (*fa*) gene on chromosome 5 (*Zucker*-*fa*/*fa*–fatty rat) have mainly been used [8]. In addition, hypercaloric diets are most frequently used to generate obese rodent models because they resemble the genesis of obesity in humans [9]. Cao et al. (2012) [10] have demonstrated that high-fat diets vary between 30–60% of calories coming from saturated fat, such as lard, beef tallow or coconut oil, while the sugar content, for which sucrose and fructose are generally used [11,12] typically comprises 60–65%. Evidence from rat studies indicates that high-fat feeding promotes obesity by elevation of body weight and adipose tissue, insulin resistance, changes in glucose metabolism, hypertension, dyslipidemia, and metabolic syndrome [11,13,14]. By contrast, high-carbohydrate diets present controversial results [10,15]. There is evidence that they result in increased body weight and adipose tissue associated with metabolic changes, similar to those observed in animals fed with hyperlipidic diets [16]. By contrast, recent studies have not shown an increase in body weight, although an increase in visceral adipose tissue, high plasma free fatty acids, and insulin resistance were identified and are connected with being metabolically obese, but of normal weight [10,15].

Another important aspect is that high calorie diets are based on cafeteria diets, which often do not accurately reproduce the population’s food profile [17]. Thus, the use of these diets may lead to nutritional imbalances in the animals, since there is variation in the composition of in natura foods, in the exact content and the lack of nutritional standardization [18]. Furthermore, the diet’s phenotype may vary distinctly among studies [9,11,12]. The majority of studies do not provide information about dietary design, food intake or pair feeding, which is rarely reported. Further, there is a lack of standardization in dietary design, content, source, and overall composition [19].

Thus, the key point regarding which hypercaloric diet type (sugar, fat, and a combination of sugar and fat) best characterizes, and as a representative model for the study of human obesity-related disorders, remain unclear. Therefore, this study was designed to investigate the effects of these dietary interventions—excess sugar or lard—or a combination of sugar and lard on metabolic disorders and cardiovascular health with long-term exposure. In addition, the study aimed to add to the development and characterization of a rodent obesity model from different hypercaloric diets to establish an appropriate model that is easily reproducible and mimics characteristics observed in the genesis of this condition in humans.

## 2. Materials and Methods

### 2.1. Animal Care

Thirty-day-old male Wistar rats (*≅*110 g) obtained from the Animal Quarters of the Federal University of Espírito Santo (Vitória, Espírito Santo, Brazil) were individually caged and subjected to different dietary regimens. The environment was maintained under a 12 h reversed light/dark cycle that started at 9:00, in a clean-air room at a constant temperature of 23 ± 3 °C and relative humidity of 60% ± 5%. All experiments and procedures were performed in accordance with the Guide for the Care and Use of Laboratory Animals published by the U.S. National Institutes of Health [20] and current Brazilian laws. The Ethics Committee of the Federal University of Espírito Santo approved the experimental protocol (CEUA-UFES 08/2016).

### 2.2. Experimental Protocol

**Figure 1:**
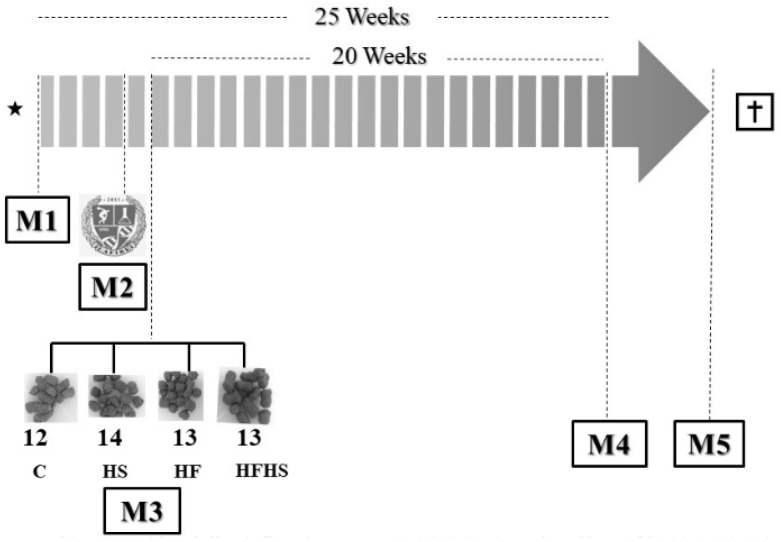
Schematic representation of the experimental design for 20 weeks. The weeks are represented by individual bars in arrow format. Moment 1 (M1): Animals’ births (Animal Quarters of the Federal University of Espírito Santo (Brazil); Moment 2 (M2): Rats were habituated to the laboratory (Experimental Laboratory of Experimental Physiology and Biochemistry—LAFIBE) and the experimenter for 7 days; Moment 3 (M3): Beginning of the experimental protocol and randomization of groups: control diet (C), high-sugar diet (HS), high-fat diet (HF), and high-fat and high-sugar diet (HFHS)—(week 0); Moment 4 (M4): *In vivo* analysis during one week (glucose tolerance test and cardiovascular profile) after 20 weeks; Moment 5 (M5): End of experimental protocol, euthanasia, and *post mortem* analysis (characterization of obesity, nutritional profile, metabolic and hormonal measurements, organ weights, and water content determination).

The rats were randomly assigned to four groups: control diet (C; *n* = 12), high-sugar diet (HS; *n* = 14), high-fat diet (HF; *n* = 13), and high-fat and high-sugar diet (HFHS; *n* = 13). The duration of the experimental protocol was 20 consecutive weeks, following which the rats were euthanized (Figure 1). All animals had free access to water and chow (40 g/day). During the experimental protocol, body weight was recorded weekly. To analyze whether dietary-induced obesity was associated with alterations in nutritional behavior, food consumption (FC) was measured daily. Calorie intake (CI) was calculated weekly by the average weekly FC × dietary energetic density [21]. Feed efficiency (FE), the ability to transform consumed calories into body weight, was determined by following the formula: mean body weight gain (g)/total calorie intake (kcal). The HS group had water supplemented with sugar (300 g/L) in alternate weeks. For the calculation of the caloric intake of the HG group, the caloric energy from the water supplemented with sugar was also quantified (1.2 kcal/mL consumed).

### 2.3. Diets

The experimental diets provided sufficient amounts of protein, vitamins, and minerals according to the Nutrient Requirements for Laboratory Animals [22]. The diets used in the current study were formulated by Nutriave Alimentos^®^ (Vitória, Espírito Santo, Brazil). The feed ingredients were blended, homogenized, and extruded (Extru-Tech Extruder, Model E-750, Sabetha, KS, USA) in the form of pellets. Then, the pellets were dried on a horizontal conveyor dryer (20 min, temperature: ±70 °C). The composition (g/kg) and nutrients for each experimental diet (%) are described in Table 1.

### 2.4. Characterization of Obesity

After 20 weeks of the experimental protocol, rats were anesthetized intraperitoneally with ketamine hydrochloride (50 mg/kg/ip, Dopalen, Sespo Indústria and Comércio Ltda., Vetbrands Division, Jacareí, São Paulo, Brazil) and xylazine hydrochloride (10 mg/kg/ip, Anasedan, Sespo Indústria and Comércio Ltda., Vetbrands Division, Jacareí, São Paulo, Brazil). Following this, their chests were opened by mid-thoracotomy, and the adipose tissue fat pads were dissected and weighed. The adiposity index used to assess obesity was calculated using the following formula: adiposity index (body fat (BF)/final body weight) × 100. BF was measured from the sum of the individual fat pad weights as follows: BF = epididymal fat + retroperitoneal fat + visceral fat.

### 2.5. Comorbidities Associated with Obesity

#### 2.5.1. Cardiovascular Profile *in vivo*

After the conclusion of the experiments (before killing), the systolic blood pressure (SBP) was measured in conscious rats using the non-invasive tail-cuff method with an electro-sphygmomanometer (IITC INC, Life Science, Woodland Hills, CA, USA). The animals were warmed in a wooden box between 38–40 °C with heat generated by two incandescent lamps for 4 min to cause vasodilation in the tail artery, and rats were then transferred to an iron cylindrical support that was specially designed to allow total exposure of the animal’s tail. After that, a sensor was placed in the proximal region of the tail, coupled to the electro-sphygmomanometer. The arterial pulsations were recorded in a computerized data acquisition system (AcqKnowledge^®^ MP100, Biopac Systems, Inc., Santa Barbara, CA, USA). The average of three readings was recorded for each measurement. Records associated with tail movement and/or other stressors that could interfere with the measurement were discarded.

#### 2.5.2. Glucose Tolerance

After 20 weeks, all rats were fasted for 6 h prior to the glucose tolerance test. After fasting, a blood sample from the tip of the tail was collected. The basal blood glucose level of each animal was immediately determined using a handheld glucometer (Accu-Chek Go Kit—Roche Diagnostic Brazil Ltda., São Paulo, Brazil). Subsequently, an injection of glucose solution (2 g/kg body weight) dissolved in water was administered intraperitoneally, and blood glucose levels were measured after 30, 60, 90, and 120 min [23]. Glucose intolerance was evaluated by the area under the curve (AUC) for glucose.

#### 2.5.3. Metabolic and Hormonal Measurements

At the end of the experimental period, the animals were subjected to 12–15 h of fasting, and blood samples were collected in dry tubes. The serum was separated by centrifugation at 10,000 rpm for 10 min. (Heraeus Megafuge 16R Centrifuge, Thermo Scientific, Massachusetts, USA) and stored at −80 °C for subsequent analysis (Coldlab Ultra Freezer CL374-86V, Piracicaba, São Paulo, Brazil). Serum glucose (GL), triglycerides (TG), total cholesterol (T-Chol), and high-intensity cholesterol (HDL) concentrations were measured using specific kits (Bioclin Bioquímica^®^, Belo Horizonte, Minas Gerais, Brazil and Synermed do Brasil Ltda., São Paulo, Brazil) and analyzed by automated biochemical equipment BS-200 (Mindray do Brasil-Comércio and Distribuição de Equipamentos Médicos Ltda., São Paulo, Brazil). Leptin and insulin levels were determined using an enzyme-linked immunosorbent assay (ELISA) using specific kits (Linco Research Inc., St. Louis, MO, USA). The reading was carried out using a microplate reader (Asys Expert Plus Microplate Reader, Cambourne, Cambridge, UK).

### 2.6. Organ Weights and Water Content

To evaluate the water content of the liver and lungs, the tissue wet weight was measured, and then the tissue was dried for 24 h at 100 °C; the dry weight was also measured. The dry weight (DW) and wet weight (WW) ratio was calculated as ((1 – DW)/WW) × 100. In addition, the absolute pancreas weight was also measured.

### 2.7. Statistical Analysis

Data from nutritional characteristics, metabolic measurements, and cardiovascular data were reported as mean ± standard error of the mean (SEM) and submitted to the Kolmogorov–Smirnov test to determine adherence to normality. Results obtained from the nutritional characteristics, metabolic measurements, and SBP were analyzed using one-way analysis of variance (ANOVA) followed by the Tukey *post hoc* test. The weekly evolution of body weight and glucose tolerance test were submitted to two-way ANOVA for the repeated measures and complemented by the Tukey *post hoc* test. The level of significance was 5%.

## 3. Results

### 3.1. General Characteristics

**Figure 2:**
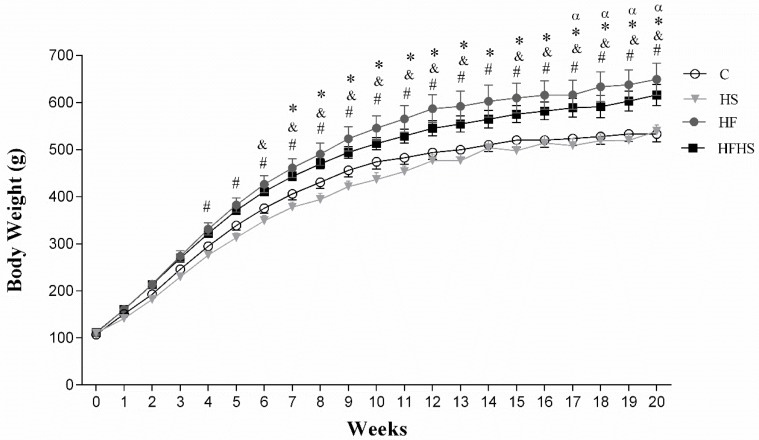
Schematic Evolution of body weight during the 20 weeks of the experimental protocol. Control diet: (C; *n* = 12), high-sugar diet (HS; *n* = 14), high-fat diet (HF; *n* = 13), and high-fat and high-sugar diet (HFHS; *n* = 13). Data are presented as the mean ± SEM. Repeated-measures two-way ANOVA for independent samples followed by Tukey *post hoc* test. *p* < 0.05. * C vs. HF; ^α^ C vs. HFHS; ^#^ HF vs. HS; ^&^ HFHS vs. HS.

The evolution of body weight during the 20 weeks of the experimental protocol is displayed in Figure 2. The change in the weekly weights of the groups was similar in the first three weeks of treatment, but after the 7th week, the body weights of the HF rats were significantly higher than those of the C rats (Figure 2), which remained significantly greater during the 20 weeks of the experiment. In addition, the HFHS group presented a significant elevation in BW when compared to C only in the last four weeks of the experimental protocol. Furthermore, the results show that HF and HFHS developed greater body weights than HS from the fourth and sixth week of treatment, respectively.

**Figure 3:**
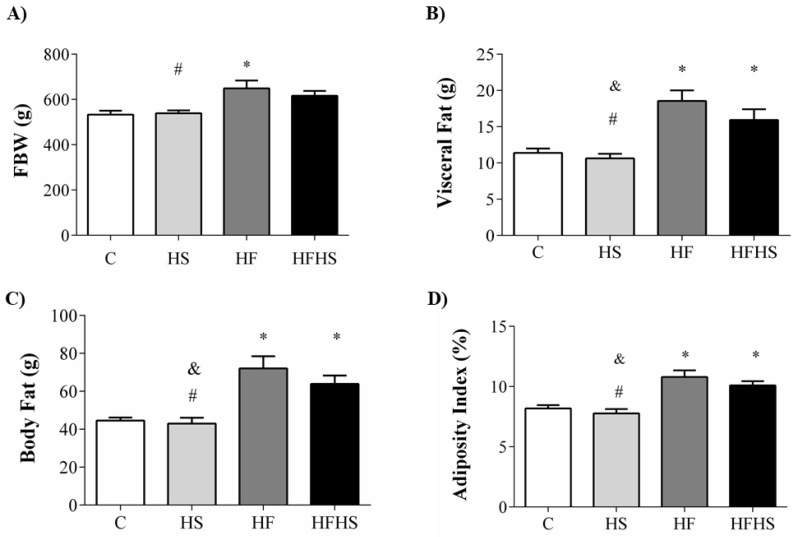
Body weights and fat mass content in rats fed either the control diet (C; *n* = 12), high-sugar diet (HS; *n* = 14), high-fat diet (HF; *n* = 13) or high-fat and high-sugar diet (HFHS; *n* = 13). (**A**) FBW = final body weight; (**B**) visceral fat pad; (**C**) BF: body fat; (**D**) AI: adiposity index. Data are presented as the mean ± SEM. One-way ANOVA for independent samples followed by Tukey *post hoc* test. *p* < 0.05 vs. * C; ^#^ HF vs. HS; ^&^ HFHS vs. HS.

After 20 weeks of the experimental protocol, rats fed with HF gained more body weight (an elevation of 22% and 20.6%) compared to those fed with control and high-sugar diets. There was no difference between C and HS for body weight gain; the same behavior was observed between HF and HFHS rats (Figure 3A). The HF and HFHS diets promoted a substantial elevation in the visceral fat pad compared to C and HS diets. Specifically, HF and HFHS rats presented an elevation of 62% and 39% when compared to C. In addition, compared to HS, the increase was 74.5% and 50% in this fat pad, respectively (Figure 3B). Corroborating these findings, HF and HFHS rats have a body fat content of 62% and 43%, respectively, and an adiposity index of 31.3% and 22.9%—percentages which are significantly greater than those for C (Figure 3C,D). In relation to HS, there was a 68% increase for HF and a 48.7% increase for HFHS in body fat, respectively. On the other hand, there were no differences in body fat and the adiposity index between C and HS rats (Figure 3C,D).

### 3.2. Nutritional Profile

**Figure 4:**
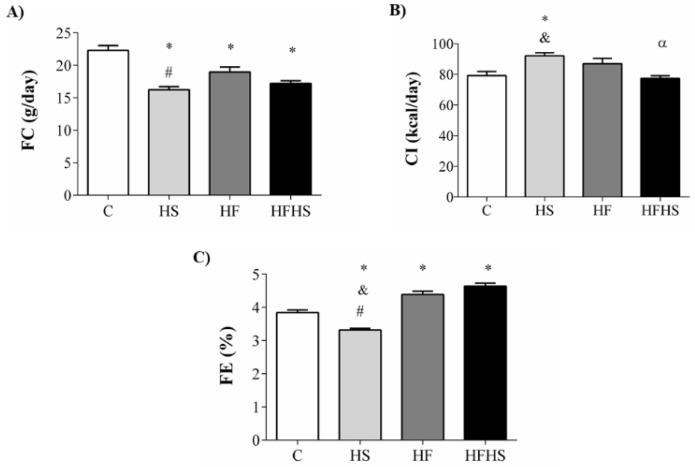
Nutritional profile of rats fed either the control diet (C; *n* = 12), high-sugar diet (HS; *n* = 14), high-fat diet (HF; *n* = 13), or high-fat and high-sugar diet (HFHS; *n* = 13). (**A**) FC = food consumption; (**B**) CI: caloric intake; (**C**) FE: feed efficiency. Data are presented as the mean ± SEM. One-way ANOVA for independent samples followed by Tukey *post hoc* test. *p* < 0.05 vs. * C; ^#^ HF vs. HS; ^&^ HFHS vs. HS; ^α^ HF vs. HFHS.

Adult male rats fed the C diet for 20 weeks had an approximately 15.2%, 22.8%, and 27.3% greater daily food consumption (g) than the HF, HFHS, and HS groups, respectively, but the daily caloric intake was higher in the HS group in relation to the C and HFHS groups (HS: 92 ± 2.0 vs. C: 79.2 ± 2.6 and HFHS: 77.3 ± 1.7 kcal/day, *p* < 0.05). In addition, there was a difference in FC between rats fed the HF diet compared with the HS group, since rats fed the HS diet consumed significantly less food (HG: 16.2 ± 0.4 vs. HF: 18.9 ± 0.7; *p* < 0.05). Furthermore, HF presented a 12.4% increase in caloric intake over HFHS (*p* < 0.05). There was no difference in the caloric intake of C compared to HF and HFHS (*p* > 0.05). While the feed efficiency (%) was higher in the HF (14.3%) and HFHS groups (20.8%) than in C (Figure 4C), the HS rats presented lower feed efficiency (FE) in relation to C rats (HS: 3.32 ± 0.05 vs. C: 3.84 ± 0.08; *p* < 0.05).

### 3.3. Comorbidities Associated with Obesity

The comorbidities associated with obesity are summarized in Table 2. HF caused significant metabolic and hormonal alterations with higher AUC and leptin levels than in C rats. In addition, AUC was elevated in the HF group compared to HS. HFHS also presented an elevation in AUC in relation to C rats. Rats fed HF, HS, and HFHS diets for 20 weeks had an approximately 13%, 17.8%, and 17.8% higher SBP, respectively, than group C (*p* < 0.05). The other parameters, including glucose, TG, T-Chol, and HDL, were similar among the groups.

### 3.4. Organ Weights and Water Content

Table 3 shows that liver weights of the HF and HFHS groups were higher than those of the C and HS groups (*p* < 0.05) and were elevated by 25%. Nevertheless, there were no differences in absolute lung and pancreas weights among the groups. Additionally, compared with C animals, there was a statistical reduction in the percentage of water in the liver in the HFHS group. Water percentages of the lungs in all rats were similar among the groups (Table 3).

## 4. Discussion

This study aimed at the development and characterization of an obesity experimental model in rodents by using dietary interventions of excess sugar, lard, or a combination of lard and sugar, which could induce an excess of adipose tissue and metabolic disorders. The HF and HFHS models share many important features with human obesity, such as increased adiposity, hypertension, glucose intolerance, and hyperleptinemia. However, a high-sugar diet did not cause obesity and metabolic disorders but only hypertension.

Experimental models that mimic the eating habits of the population have been widely used to elucidate the mechanisms of obesity and metabolic disorders [24]. In this sense, several studies use diets with added food and/or hypercaloric ingredients; however, it is not always possible to see a balance between nutritional components, and the conclusions may be due to the increase of a specific component with a reduction in the others [9,11,25]. Studies have demonstrated that the fat percentage varies between 20–60% and, generally, can be of either animal origin (lard and fish oil) or vegetable (olive oil and coconut oil) [11]. In relation to dietary approaches with carbohydrates, this macronutrient represents 60–70%, and the simple carbohydrate sources most frequently utilized are sucrose and fructose, respectively [9,12]. In accordance with the existing research, in the current study, lard was used as a source of fat; the percentage of this macronutrient was 37.6% and 37.4% in the HF and HFHS diets, respectively. For HS, sugar was used as the source of simple carbohydrates plus water with sugar, representing 58% of the carbohydrates. It should be noted that in the hypercaloric dietary interventions used, lard and sugar were added from a reduction of inert material present in the chow for the purpose of balancing the concentration of the ingredients.

The weekly monitoring of body weight aimed to identify the moment of significant increase in this parameter in animals under the influence of treatments with hypercaloric diets in relation to those treated with a standard diet to effectively evidence the duration of obesity from a given experimental model [26,27]. In this context, the HF diet promoted a significant increase in body weight in relation to the animals fed a control diet (C) from the seventh week until the end of treatment (20th week). These findings are in agreement with the literature, in which it is evidenced that the body weight gain occurs gradually during the feeding period with an excess of fat [28,29]. One important aspect is that although it is possible to note or evidence the difference in body weight after two weeks of the experimental protocol, the obesity phenotype becomes more apparent after prolonged periods, generally after four weeks [11,26,30]. Corroborating this information, the HFHS showed an elevation in body weight only in the last 4 weeks of the experimental protocol. In contrast to other studies [16], the current experimental model of a high-sugar diet did not promote a substantial elevation in body weight during the 20 weeks of the experimental protocol.

According to the literature, fat-enriched diets have been used for decades to model obesity in rodents [31]. The development of obesity from a HF diet was characterized by significant differences in the percentage of BW gain and the adiposity index in relation to the C diet; they were elevated by 22% and 62%, respectively. Consistent with previous investigations, hyperlipidic diets that used lard as a fat source evidenced an increase in body weight of about 10–15%, while the adiposity increase was 30–45% [5,32,33]. Thus, the elevation in adipose tissue was about three times greater than the increase in body weight; these results are in agreement with our findings. Although our results have shown the development of obesity in the HF and HFHS, the measurement of adiposity was evaluated by the adipose tissue fat pads, which were dissected and weighed. Thus, to support fat mass measurements, further research is warranted to investigate the histological analysis for the purpose of quantifying the size of adipocytes and confirm our findings. Physiologically, obesity is also related to increases in levels and volume of adipocytes [34]. During adipogenesis, a cascade of transcription factors and adipocyte-specific gene expression lead to adipocyte development [35,36], which is regulated by peroxisome proliferator-activated receptor gamma (PPARγ) and CCAAT/enhancer binding proteins, C/EBP (C/EBP-α) [36,37,38]. However, in the current study, we did not measure the levels of these regulators of adipogenesis

The literature shows that excess fat associated with sugar promotes elevated body weight gain [16,39], but in disagreement with the initial hypothesis of this study, HFHS did not cause a significant increase in FBW. This dietary intervention, however, resulted in an increase in visceral and retroperitoneal fat deposits, as well as in the adiposity index. This finding corroborates studies that have verified an increase in the prevalence of metabolically obese individuals without changes in their body weight, mainly characterized by an increase in adipose tissue, especially in the abdominal region [10,40]. Other studies have shown that HFHS consumption does change FBW, but promotes an increase in adipose tissue [15,41,42]. These data allow us to conclude that the HFHS promotes obesity by increasing adipose tissue without alterations to body weight.

The elevation in body weight and adiposity in the hyperlipidic experimental models increased with sugar, refers to caloric intake and food efficiency which, generally, lead to a greater availability of calories and hyperphagia [1,43]. Even without an increase in caloric intake, it is possible to develop obesity, since the change in nutrient composition influences the efficiency of food utilization, thus leading to an increase in fat storage per calorie consumed. The elevation in body fat may be due to an increase in the caloric density of the diet, which may lead to a higher total caloric intake or an increase in the intake of a given macronutrient [44]. Although they had a similar total caloric intake, the HF and HFHS groups consumed 61% and 43% more calories from fat, respectively, than the C animals did.

Interestingly, HS did not lead to an increase in body weight and adipose tissue other than in C. The literature is controversial regarding the experimental obesity models from excessive sugar [10,15,16,45]. Malafaia et al. (2013) [16] observed an elevation in the body weight of animals receiving a 30% sucrose diet from the second week on of the experimental protocol. However, Cao et al. (2012) [10] observed that Sprague Dawley rats fed 35% of their dietary calories from sugar for 20 weeks had similar body weights; however, there was an increase in adipose tissue. Despite the higher caloric intake attributed to the consumption of water with sugar, food consumption and feed efficiency were lower than for the C group. According to Sclafani [46], experimental models that receive sugary drinks increase energy consumption by around 10–20%. In the current study, the caloric intake was 16% higher in the HS group. Our findings corroborate those of Castellanos Jankiewicz et al. (2016) [47] and Sheludiakova et al. (2011) [48], who also reported a higher caloric intake in the form of simple sugars, but without alterations to body weight, suggesting a lower feed efficiency. Obesity-resistant animals usually present metabolic alterations that suppress lipogenesis and accelerate β-oxidation, contributing to the antiobesogenic effect observed in these animals [49]. Thus, the current experimental model from high-sugar could present increased expression of some enzymes associated to fatty acid β-oxidation and reduced expression of enzymes involved in lipogenesis, such as carnitine palmitoyltransferase 1, and fatty acid synthase and acetyl-CoA carboxylase, favoring the fat over carbohydrate oxidation in muscle skeletal [49]. Nevertheless, these parameters were not evaluated in this study.

Obesity has been associated with several comorbidities, such as glucose intolerance, hyperinsulinemia, insulin resistance, dyslipidemia, and hypertension [11,13]. The HF and HFHS obesity experimental models also presented almost all the metabolic disorders, such as glucose intolerance visualized by insulin resistance; however, elevations in fasting glycemia, hypertriglyceridemia, hypercholesterolemia, and reduced HDL were not observed. These results are divergent from other findings [14,15,45,47,48,50,51]. In addition, the mechanism(s) by which a diet high in saturated fat and sugars, as well as fat combined with sugar, induce changes in the glucose profile, are related to a reduction in the number of insulin receptors and the activity of the glucose transport system, as well as to the intercellular metabolism of glucose [52].

The findings of the present study also demonstrate that only the HL group developed hyperleptinemia, since the animals had 106% more leptin than those of C. However, these animals did not become hyperphagic, thus suggesting that they were not resistant to leptin—a metabolic disorder frequently observed in obesity. According to Knight et al. [53], in the early stages of obesity, there is the development of peripheral leptin resistance, attributed mainly to the saturation of the leptin transport system through the blood–brain barrier. However, after long-term exposure to a high-fat diet (>20 weeks), animals become resistant to leptin, even when it is infused directly into the brain, thus suggesting that in these animals, the leptin receptors lack the ability to activate leptin receptor signaling pathways [53].

In addition to changes in glucose and biochemical and hormonal homeostasis, obesity is also a risk factor for the development of hypertension. In this sense, the models proposed in the current study led to an elevation in blood pressure levels. According to Lim et al. [54], there is a strong correlation between body weight gain and an increase in blood pressure. The accumulation of adipose tissue raises the demand for oxygen and increases blood volume and cardiac output. In hyperglycemic experimental models, even without an association with obesity, hypertension is also reported in the literature [55,56].

Another important aspect observed in the current study was an elevation in the percentage of liver water content in an HFHS liver in relation to that of C, even with a similar total weight. This suggests fat accumulation in this tissue, resulting mainly from the elevation of free fatty acids which, when excessive, can be synthesized in TG and deposited in the liver [50]. Nevertheless, our results need to be strengthened by further studies, since the changes observed in liver of the HF and HFHS groups were only determined by weight, without oil-red O staining analysis to support our findings.

## 5. Conclusions

In conclusion, our study shows that approaches employing an excess of lard, and a combination of sugar and lard, promote obesity and its comorbidities. These approaches represent useful tools for studying human obesity-related disorders. An excess of sugar, however, did not present these characteristics, thus proving not to be a good model for obesity.

## Figures and Tables

**Table 1:** Composition and nutritional values of diets used in the experiment.

Components (g/kg)	Diets			
	C	HS *	HF	HFHS
Corn	200	200	180	80
Rice	200	200	200	200
Bone meal	120	120	120	120
Sugar	-	100	-	100
Soy oil	75	75	-	-
Lard	-	-	200	200
Gluten	200	200	200	200
Salt	3.5	3.5	3.5	3.5
Mineral Mix **	35	35	30	30
Vitamin Mix **	16.5	16.5	16.5	16.5
Inert Material ***	150	50	50	50
**Total (g)**	1000	1000	1000	1000
**Nutrient Composition (%)**				
Protein	24.8	21.8	17.8	19.2
Carbohydrate	49.6	52.3	44.6	43.4
Lipids	25.6	25.9	37.6	37.4
**Energy Density (Kcal/g)**	3.55	3.65	4.59	4.49

Diets. C: normal rodent chow; HS: high-sugar; HF: high-fat; HFHS: high-fat and high-sugar. * Rats received a diet of simple carbohydrates and water supplemented with sugar (300 g/L) during alternate weeks. In order to calculate the caloric intake of HS, the caloric value of the sugar diet (3.65 kcal/g) plus the caloric value of water intake with sugar (1.2 kcal/mL) was computed. ** Vitamin and Mineral Mix: vit. A, vit. C., vit. D3, vit. E, vit. K3, vit. Complex B, pantothenic acid, folic acid, biotin, choline; selenium, iron, copper, manganese, iodine, zinc, cobalt, calcium, and phosphorus. *** Bentonite: inert material, with no nutritional value and calories.

**Table 2:** Comorbidities and hormones associated with experimental diets.

Variables	Experimental Groups				
	C	HS	HF	HFHS	*P* Value
**Glucose (mg/dL)**	108 ± 2	112 ± 3	115 ± 4	115 ± 3	0.26
**AUC (mg/dL/min)**	1234 ± 57	1341 ± 29	1568 ± 71 *^#^	1478 ± 41 *	0.008
**SBP (mmHg)**	146 ± 3	172 ± 4 *	165 ± 3 *	172 ± 2 *	0.001
**TG (mg/dL)**	35 ± 7	29 ± 5	50 ± 6	36 ± 7	0.08
**T-Chol (mg/dL)**	83 ± 4	73 ± 3	81 ± 5	73 ± 3	0.13
**HDL (mg/dL)**	27 ± 1	24 ± 1	29 ± 3	25 ± 1	0.12
**Leptin ¹ (ng/mL)**	12 ± 3	13 ± 4	25 ± 3 *	17 ± 4	0.03

Values are means ± SEM. Control (C; *n* = 10), high-sugar (HS; *n* = 14), high-fat diet (HF; *n* = 12) or high-fat and sugar diet (HFHS; *n* = 11). ^1^ Leptin determination: (*n* = 6) *n* = number of animals. AUC: area under the curve for glucose; HOMA-IR: homeostatic model assessment index; SBP: systolic blood pressure; TG: triglycerides; T-Chol: total cholesterol; HDL: high-density lipoprotein. One-way ANOVA for independent samples and Tukey *post hoc* analysis. *p* < 0.05 vs. * C; ^#^ HF vs. HS.

**Table 3:** Organ weights and water contents.

Organ	Experimental Groups				
	C	HS	HF	HFHS	*P* Value
**Liver (g)**	13.4 ± 0.6	14.2 ± 0.5	16.8 ± 0.7 *^#^	16.9 ± 0.8 *^&^	0.001
**Lung (g)**	1.86 ± 0.10	1.85 ± 0.08	1.95 ± 0.05	1.92 ± 0.07	0.77
**Pancreas (g)**	0.94 ± 0.06	1.15 ± 0.06	1.00 ± 0.08	1.08 ± 0.04	0.08
**Liver (%)**	68.7 ± 0.2	66.8 ± 0.7	66.7 ± 0.4	66.5 ± 0.7 *	0.04
**Lung (%)**	79.1 ± 0.4	79.1 ± 0.6	79.3 ± 0.5	78.5 ± 0.6	0.71

Values are means ± SEM. Control (C; *n* = 12), high-sugar (HS; *n* = 14), high-fat diet (HF; *n* = 13), or high-fat and sugar diet (HFHS; *n* = 13). One-way ANOVA for independent samples and Tukey *post hoc* analysis. *p* < 0.05 vs. * C; ^#^ HF vs. HS; ^&^ HFHS vs. HS.
